# Understanding variations in catastrophic health expenditure, its underlying determinants and impoverishment in Sub-Saharan African countries: a scoping review

**DOI:** 10.1186/s13643-018-0799-1

**Published:** 2018-09-11

**Authors:** Purity Njagi, Jelena Arsenijevic, Wim Groot

**Affiliations:** 1 United Nations University - Maastricht Economic and social Research institute on Innovation and Technology(UNU-MERIT), Maastricht University, Maastricht, The Netherlands; 2 Department of Health Services Research, Faculty of Health Medicine and Life Sciences, Maastricht University, Maastricht, The Netherlands; 3 Faculty of Law, Economics and Governance, Utrecht University, Utrecht, The Netherlands

**Keywords:** Catastrophic health expenditure, Impoverishment, Out of pocket payments, Sub-Saharan Africa, Scoping review

## Abstract

**Background:**

To assess the financial burden due to out of pocket (OOP) payments, two mutually exclusive approaches have been used: catastrophic health expenditure (CHE) and impoverishment. Sub-Saharan African (SSA) countries primarily rely on OOP and are thus challenged with providing financial protection to the populations. To understand the variations in CHE and impoverishment in SSA, and the underlying determinants of CHE, a scoping review of the existing evidence was conducted.

**Methods:**

This review is guided by Arksey and O’Malley scoping review framework. A search was conducted in several databases including PubMed, EBSCO (EconLit, PsychoInfo, CINAHL), Web of Science, Jstor and virtual libraries of the World Health Organizations (WHO) and the World Bank. The primary outcome of interest was catastrophic health expenditure/impoverishment, while the secondary outcome was the associated risk factors.

**Results:**

Thirty-four (34) studies that met the inclusion criteria were fully assessed. CHE was higher amongst West African countries and amongst patients receiving treatment for HIV/ART, TB, malaria and chronic illnesses. Risk factors associated with CHE included household economic status, type of health provider, socio-demographic characteristics of household members, type of illness, social insurance schemes, geographical location and household size/composition. The proportion of households that are impoverished has increased over time across countries and also within the countries.

**Conclusion:**

This review demonstrated that CHE/impoverishment is pervasive in SSA, and the magnitude varies across and within countries and over time. Socio-economic factors are seen to drive CHE with the poor being the most affected, and they vary across countries. This calls for intensifying health policies and financing structures in SSA, to provide equitable access to all populations especially the most poor and vulnerable. There is a need to innovate and draw lessons from the ‘informal’ social networks/schemes as they are reported to be more effective in cushioning the financial burden.

**Electronic supplementary material:**

The online version of this article (10.1186/s13643-018-0799-1) contains supplementary material, which is available to authorized users.

## Background

Financial barriers are a key limitation to access health services in low- and middle-income countries (LMICs) [1, 2]. Financial barriers are usually related to out of pocket patient payments and their impact on household budget [3]. Two main approaches are used to assess the financial barriers: catastrophic health expenditure that occurs when out of pocket (OOP) payment equals or exceeds a pre-specified threshold of household expenditure or capacity to pay [4, 5], and impoverishment that occurs when the average household consumption after health care payment is below the pre-specified international or national poverty line [6].

The incidence of catastrophic payments is reported to be higher in low-income countries that rely on OOP, and lower in countries that have some prepayment mechanisms [5, 7]. While impoverishment is usually reported in LMICs, catastrophic payments also exist in high-income countries, and are slightly concentrated amongst the less well-off [5, 8]. This trend is also observed in African countries. Studies in Sub-Saharan African (SSA) countries have shown that inequities in access exist as a result of income differences and the level of OOP within the country. The proportion of households facing catastrophic health care payments has been shown to vary widely between countries [9–11].The World Health organization (WHO) argues that when people suffer financial hardship due to OOP, it is impossible to get closer to universal health coverage (UHC) due to the high risk of catastrophe and impoverishment [12]. Moreover, UHC aims to ensure that health care benefits are distributed on the basis of need for care and not on ability to pay [13]. The burden of OOP payments has encouraged SSA countries to use different financial arrangements to prevent catastrophic payments [14]. One of them is introduction of insurance systems with universal population coverage [15], and another is removal of user fees. There is also a trend by governments to move out of the OOP payments that are considered to impoverish those who are already poor [16]. Given the over reliance on OOP payments in most of SSA countries, and with most countries having inadequate social insurance schemes, there is a strong need to evaluate systematically the existing evidence on financial inequity in access to healthcare. Furthermore, the effectiveness of the health financing system is seen through protecting people against the risk of becoming poor, while enabling them to make use of services [17]. In addition, WHO underscores health care financing as one of the crucial components of the broader efforts to ensure social protections [12].

Systematic reviews synthesise evidence given their clearly formulated structure and the methodological rigour [18]. Several studies have reviewed the variations in health indicators in SSA; however, most of these have focused on health status, service coverage and utilisation indicators like mortality rates and incidence/prevalence of diseases [19, 20]. There have been few reviews that focus on unequal access to health care due to financial barriers [21]. Lack of systematic reviews in this area is perceived by policy makers as a limitation in decision making and developing new strategies [22]. While there are several systematic reviews on catastrophic payments and impoverishment in LMICs [23, 24], very few have incorporated literature from SSA, and those that have done so have included only one or two countries from the SSA region [5, 25]. A few other reviews that have been conducted are disease specific [26–28] and do not review the CHE risk factors. To our knowledge, there is currently no systematic and/or scoping review that examines the scale and variations of CHE and impoverishment across SSA countries. To understand the scope and nature of the studies conducted in SSA, we apply a scoping review approach. Scoping reviews are considered appropriate in that they not only bring together the available evidence but also provide broader synthesis of the evidence [29]. The aim of this study is to provide an overview of the magnitude and distribution of catastrophic health expenditure and impoverishment due to OOP for healthcare across SSA countries. Furthermore, we also look into the determinants of CHE that have been identified across SSA countries. This will not only highlight the scale of the problem but also identify any gaps that could potentially strengthen future research in CHE/impoverishment in SSA countries. The findings will help in developing effective health policies [30], that are more targeted, prioritise the vulnerable populations and address key risk factors. In addition, the findings could help to inform strategic health financing priorities of SSA member states by development partners/regional blocs like the African union (AU), WHO and World bank amongst others that invest in health initiatives in the region.

This study therefore responds to the research question: what variations exist in the distribution of CHE and/or impoverishment and the associated risk factors across SSA countries? The paper continues with the methods section (searching strategy and study selection) followed by the results section and discussion with conclusions.

## Methods

This scoping review is based on the framework proposed by Arksey and O’Malley [31] and incorporates recommendations proposed by Levac [32]. In addition, the Preferred Reporting Items for Systematic Reviews and Meta-Analyses [PRISMA] (See Additional file 1) that provides key items considered to be essential and minimum components of a systematic review or meta-analysis protocol [33], was applied to guide the screening and eligibility of the studies.

### Search strategy and inclusion criteria

#### Inclusion and exclusion criteria

This review included studies that focused on all population groups including vulnerable groups like people living with disability, the elderly or children in both rural and urban settings. Studies with the primary aim of assessing catastrophic health expenditure and household impoverishment due to out of pocket payments in health care were included. We particularly look at the incidence of CHE and impoverishment, defined as the proportion of households whose out of pocket spending on health care is catastrophic or drives them into poverty. The intensity of CHE or impoverishment defined as the extent to which the household expenditure exceeds the set threshold or poverty line was also included. In addition, we reviewed studies that assessed the risk factors associated with the observed levels of incidence in catastrophic health expenditure.

We considered studies that assessed CHE and/or impoverishment due to seeking any type of health care service including HIV/AIDS, TB, chronic illnesses, malaria and maternal health services. The review was restricted to studies undertaken in any of the 45 Sub-Saharan African countries with coverage of either part of the country, the entire country or multiple countries. Articles were considered for inclusion if they were observational studies including cross-sectional studies, case-control, comparative or longitudinal studies. We excluded articles that were discussion papers or general literature review on CHE or impoverishment, qualitative studies that discussed CHE and those that addressed methodological issues and global macro analysis on CHE. These articles do not provide outcome measures that are relevant for our study such as the incidence or intensity of CHE.

### Search strategy

We commenced with a general search on Google Scholar, and then searched in several databases namely PubMed, EBSCO (EconLit, PsychoInfo, CINAHL), Web of Science and Jstor. We also searched through the grey literature of relevant organisations virtual libraries such as World Health Organization (WHO) and the World Bank. In addition, a forward search of authors mentioned in selected articles was also conducted. The search terms included ‘Catastrophic’, ‘Impoverishment’, ‘Financial burden’, ‘Economic burden’, and under PubMed search, we included the MESH terms for health expenditure, health care costs and Sub-Saharan African countries. These words were used for all the other database searches. The detailed search chain for PubMed is provided in Additional file 2. Only studies published in English language in the last 10 years (2006–May 2017) were included for review.

### Data extraction and analysis

The main reviewer extracted and analysed data from all articles in consultation with the other authors. Information extracted from the publications included context of the study (country and year of publication), characteristics of the included population, methodology (design of the study, data source, sample size, type of analysis), primary (incidence and intensity of CHE/Impoverishment) and secondary outcomes (determinants of CHE). Studies were grouped by the outcome measures. As a primary outcome measure, we use the incidence and intensity of catastrophic expenditure and impoverishment. To measure the impact of OOP on household expenditure; varying thresholds were applied which varied from 5 to 40% as a ratio of household expenditure or non-food expenditure. Information on the determinants of CHE and impoverishment were reported as secondary outcomes. Articles were also classified according to four major SSA regions (West Africa, East Africa, South Africa and Central Africa).

### Quality and risk of bias assessment

Although quality assessments are not a standard requirement in scoping reviews, it has been argued that the lack of it could minimise the rigour and challenge the interpretation of the findings [32]. In light of this, quality assessment of the studies was conducted by the main reviewer in consultation with the other authors. The Quality Assessment Tool for Observational Cohort and Cross-Sectional Studies was applied to evaluate the quality of the studies included. The tool is recommended by the NIH and has been used in several systematic reviews to assess internal validity [34] (See Additional file 3). For each question, studies are given scores on a Yes (1) or No (0), and others which include CD, cannot determine; NA, not applicable and NR, not reported.

**Fig. 1:**
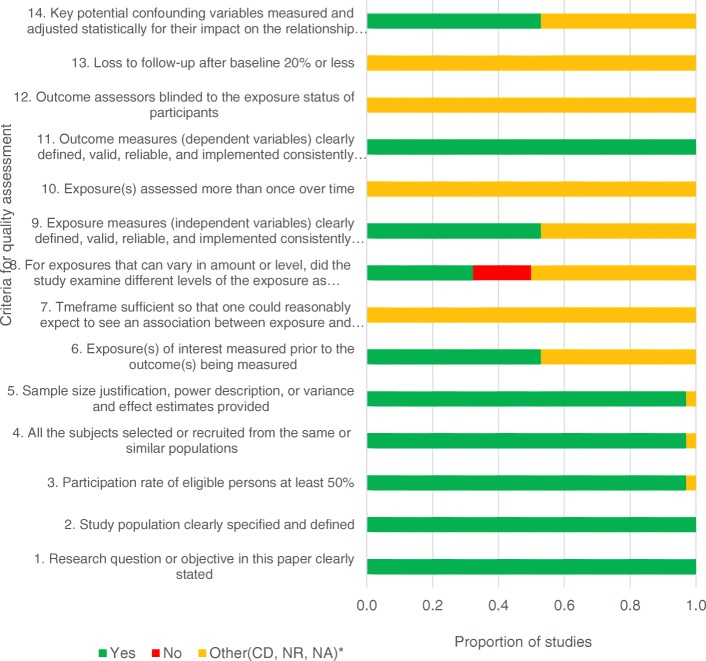
Quality assessment of included studies. The figure represents a summary of the quality assessment scores as per the assessment criteria/checklist

All the studies included in this review were assessed for quality using the criteria that fits the respective studies, and for those studies which some elements of the criteria did not apply, these were marked as not applicable. The assessment of exposure measures was only done for those studies that focused on the risk factors associated with CHE. On average, all the studies met the quality criteria, apart from six studies [11, 13, 24, 29, 31, 34] that assessed exposure factors that vary by levels that did not examine the different levels of exposure. In addition, one multi-country study [18] did not report on the sample size; thus, the three criteria related to the study sample could not be determined. Figure 1 shows the ratio of studies that met the respective criteria.

## Results

### Study selection

**Fig. 2:**
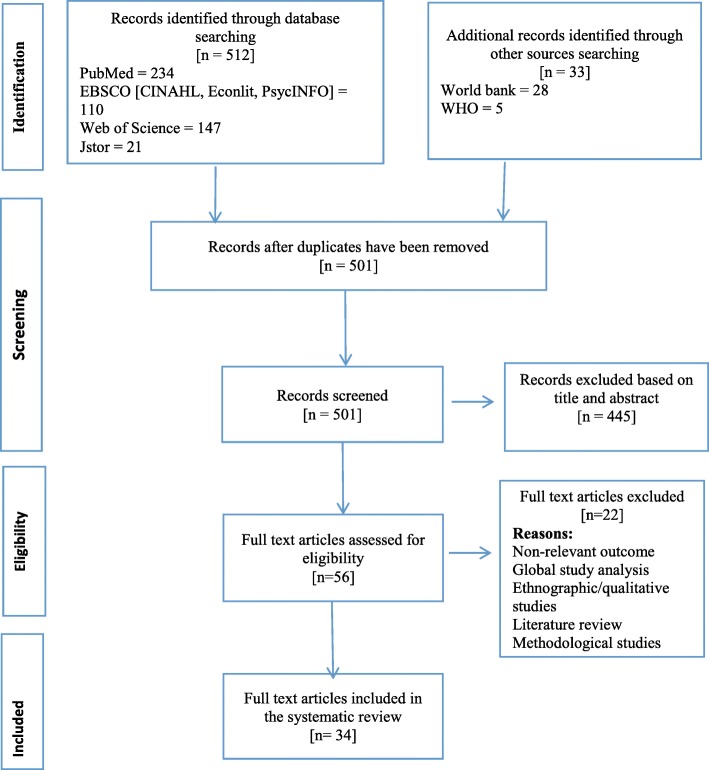
PRISMA flow chart. The figure presents the flow of information through the different phases of studies selection. It maps out the number of records identified, included and excluded, and the reasons for exclusions

The initial search identified a total of 512 articles from the main journals and another 33 articles from the additional databases of the WHO and the World Bank. Once duplicates were removed, a total of 501 articles remained. Using title and abstracts, one reviewer screened all the identified articles based on an agreed inclusion criteria with the other two authors. A total of 445 articles were excluded largely due to being non-SSA specific, or for having a general focus on national health expenditure instead of CHE and impoverishment. A total of 56 articles remained that were fully assessed for eligibility; a second reviewer went through these selected articles and provided recommendations. The three reviewers had concurrence to include 34 articles in the final review analysis. The main reason for dropping 22 studies included the fact that the outcome was level of OOP and not the proportion that was catastrophic. Also, these articles do not provide information that allow us to calculate the proportion of OOP that is/was catastrophic for households, a global analysis of studies that included one or two SSA countries, discussion papers or general literature review that provide a general understanding of CHE, qualitative studies that discussed CHE and methodological studies. Figure 2 represents the PRISMA flow chart for the studies selection process.

### Characteristics of the included studies and quality of data

Of the 34 studies assessed, half were from the West African region (11 from Nigeria), eight from the East African region (4 from Kenya), seven from the South African region (2 from South Africa and 2 that were comparative of South Africa with Lesotho and Mozambique respectively), one from the Central African region and one covered three SSA regions (East, West and South Africa). One could argue that literature from the Central African region was missing because the region is largely francophone, while the review focused only on English studies. However, there were several studies included from other French speaking countries including DRC, Burkina Faso, Mali, Benin, Senegal and Côte d’Ivoire. All the studies identified were observational, of which 27 were cross-sectional studies, 3 were cross-sectional comparative across countries in the regions, 2 were modelled longitudinal, 1 was a case control and another a prospective observational study.

Nineteen (19) of the studies focused on general health care, while 15 focused on diagnostic categories including 4 on chronic (non-communicable diseases), 5 on HIV/ART care and treatment (with one being comparison with Obstetric and TB), 2 on obstetric care (one being a comparison with TB, HIV/ART), 3 on TB (one comparison with obstetric and HIV/ART), and 3 on malaria. The number of studies increased over years with only 8 (24%) being published between 2006 and 2011, while 26 studies (76%) were published between 2012 and May 2017. Half of the studies covered a sub-national population within the respective country, while 14 studies (41%) had a national coverage, and 3 studies were multi-county in that they focused on more than one country. The national studies utilised data from various national household surveys including the National Living Standard, Social Economic Survey, Poverty Monitoring Survey, Health Expenditure and Utilization Survey, while the sub national studies sampled the respective regions or specific target population. A few other studies utilised hospital data to gather data on expenses paid for the various services provided; the limitation was the small sample sizes. See Appendix 1 for all studies included in the review by various characteristics.

### Incidence and intensity of catastrophic health expenditure in SSA countries

The large majority of studies focused only on the incidence of CHE (*n* = 23), while some focused on both incidence and intensity (*n* = 11), and a set of others focused on the determinants of CHE (*n* = 18). Catastrophic health expenditure varied greatly between countries. However, cross-country comparisons are difficult because of the different thresholds, sample sizes and data sets used in the various studies. Given these variations, we shall discuss the magnitude and distribution of catastrophic payments based on the most commonly used thresholds; that is 10% of household income [35] and 40% of non-food expenditure [9]. Table 1 below summarizes the incidence and intensity of CHE as repoted in the articles reviewed.

**Table 1:** The incidence and intensity of CHE in SSA by regions

Region (countries)	Articles that reported CHE; *n* (%)	CHE incidence: % range [threshold]		CHE intensity; % range [threshold]	
		General health care	Diagnostics^1^	General health care	Diagnostics^2^
Region 1: West Africa countries: Benin = 1; Burkina Faso = 1; Côte d’Ivoire = 1; Mali = 1; Nigeria = 11; Senegal = 1; Ghana = 1	17 (50%) [2, 4, 5, 7, 10, 11, 15–17, 19–21, 24, 26, 28, 30, 33]	2.4–25.4[*] 1.7–27[**]	8.2–71.8[*] 9.8–44[**]	3.4–7.8[*]	6–7.8 [*] 8.3 [**]
Region 2: East Africa countries: Kenya = 4; Uganda = 2; Tanzania = 1	8 (24%) [1, 3, 6, 8, 9, 13, 31, 34]	1.5–22.8[*] 2.9–18[**]	None	2.5–11[*] 5.7–25[**]	None
Region 3: South African countries: South Africa = 2; Zambia = 1; Malawi = 2; Madagascar = 1; Botswana = 1; Lesotho = 1; Mozambique = 1	7 (21%) [12, 14, 22, 23, 25, 29, 32]	0.09–11.2[*] 0.7–9.3[**]	9–39.9[*] 4.5–34[**]	1.01[*] 0.1[**]	None
Region 4: Central Africa countries: Democratic Republic of Congo = 1	1 (3%) [27]	None	46.4[*] 81.1 [**]	None	None
Region 5: Multi-region South Africa, Ghana, Tanzania = 1	1 (3%) [18]	0.1–2.4[**]	None	None	None

*At 10% household income

**At 40% non-food expenditure

^1,2^Malaria, HIV/ART, epilepsy, diabetes, TB, obstetric care

The proportion of households facing catastrophic payments varied widely by the threshold applied. In most of the studies, the incidence of catastrophic expenditure was seen to be lower when higher thresholds were applied [36, 37], at 10% the average incidence of CHE was 23% while at 40% the average was 17%. Generally, we noted that CHE was highest when a specific diagnostic service was assessed. Amongst the various diagnostics, HIV/ART and malaria had the highest incidence. Inpatient HIV patients in Nigeria had the highest incidence of CHE (100%) at 10% household expenditure, while at 40% non-food expenditure, the incidence was reduced to 94.3% [38]. There was a high incidence of CHE at 40% non-food expenditure in the Democratic republic of Congo amongst hospitalised children with severe malaria which was at 81.1% capacity to pay [39]. Both studies with a high incidence targeted specific groups of patients and thus were not national representative surveys. TB patients also incurred a high incidence of CHE in Benin at 71.8% at the threshold of 10% of household expenditure [40].

**Fig. 3:**
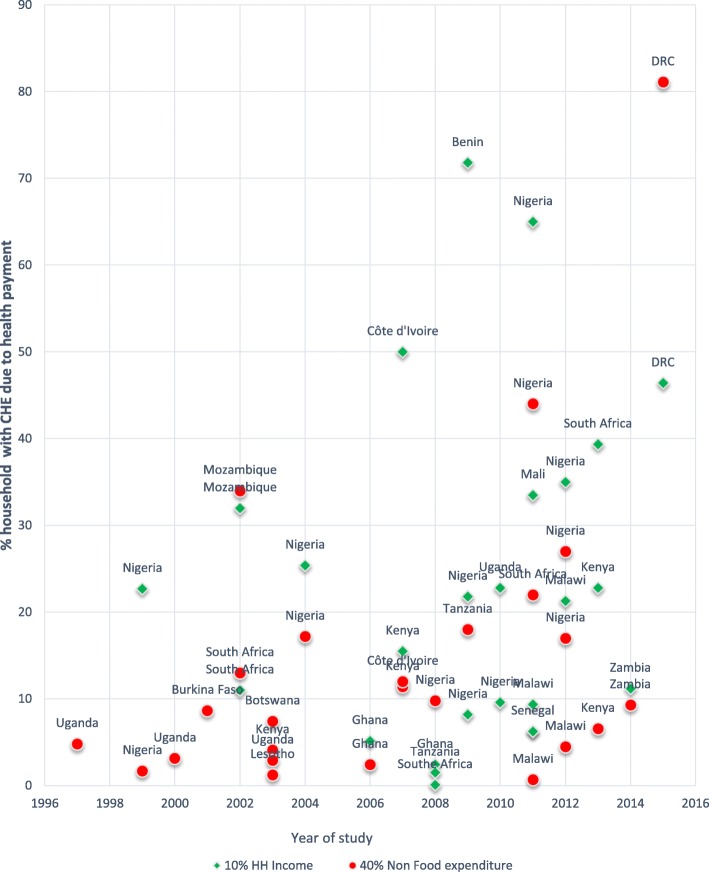
The level of CHE in SSA. The figure represents the level of CHE reported in the various studies/articles across countries over time. It plots the percentage of households with CHE at thresholds of 10% household income and 40% non-food expenditure, against the study period

Variations are also observed within countries, for instance, two national studies in Nigeria that focused on CHE at 40% of non-food expenditure, one reported CHE of 1.7% [41], while the other [42] reported ten times more at 17.2%. However, in the same studies, at 10% of household expenditure, CHE was closer in range at 22.7% and 25.7% respectively. Figure 3 shows the variations in the level of incidence of CHE in various Sub-Saharan countries, with many countries still experiencing high CHE over time.

The intensity of catastrophic health expenditure, which is an indication of how much expenditure exceeds the thresholds ranged from 0.1 to 25% when the 40% proportion of non-food household expenditure threshold is applied, and 1–11% when the 10% proportion of household expenditure is applied. There was relatively little difference between the intensity reported for general health care and that reported for specific diagnostics like HIV/ART, TB, malaria and chronic illnesses. Most studies on diagnostic care did not report on intensity; thus, we have less evidence to discuss the severity of CHE due to the use of diagnostic services and treatment. The intensity of CHE was found to be lowest amongst South African countries, while in East Africa and West Africa, the intensity was within the same range.

### Determinants of catastrophic health expenditure

Eighteen (18) studies assessed the determinants of CHE in the respective countries for both general health care and specific diagnoses. The articles assessed various determinants; thus, this review will discuss the overarching determinants reported in the majority of studies. These are summarised in Table 2. See Appendix 2 for determinants reported in each of the study.

**Table 2:** Determinants of CHE

Determinants of catastrophic health expenditure (CHE)	Western Africa	South Africa	East and Central Africa	Total studies per determinant
Household economic status: Poor-income households Middle-income households High-income households	4 [5, 15, 28, 33]	2 [12, 14]	4 [1, 6, 13, 27*]	10
Type of health care provider: Private Public	2 [11, 24]	1 [14]	5 [1, 9, 13, 27*, 34]	8
Type of illness: HIV-ART Tuberculosis Obstetrics Malaria Chronic illnesses	5 [5, 10, 11, 24, 33]	2 [12, 29]	5 [1, 6, 9, 13, 27*]	12
Household member characteristics Employment status Education level Gender/sex Age of household members	5 [5, 15, 24, 28, 33]	3 [12, 22, 29]	7 [1, 6, 9, 13, 27*, 31, 34]	15
Geographical location Distance to the health facility Residence (rural/urban)	3 [24, 11, 15]	3 [14, 22, 29]	5 [1, 6, 9, 31, 34]	11
Social insurance/health scheme Health insurance Social network scheme	3 [5, 11, 28]	Nil	1 [9]	4
Household size and composition Number of household members Household with elderly people Household with under 5 children	5 [5, 10,11, 24, 33]	2 [12, 22]	6 [1, 6, 9, 13, 31, 34]	13

*Central African region (Democratic Republic of Congo)

#### Household economic/income status

Households’ income level is the most consistent determinant of catastrophic health expenditure with higher-income groups being less likely to incur CHE relative to middle income- and lower-income groups [39, 43, 44]. This is also observed amongst HIV/ART related studies. Lower-income groups had a higher likelihood of incurring CHE on ART services, given that they are more likely to use their savings on food and other routine household expenditures [45]. Besides the type of disease, the power of association varied by country of study. For instance, amongst studies conducted on CHE due to tuberculosis (TB), it was found that the power of association (CHE–lower-income groups) was higher in Benin [40] than in Nigeria [46].

#### Type of health care provider

In case health services are provided by public hospitals, a higher CHE is observed. This is especially observed for inpatient services [47, 48]. Within the public health care system, seeking services at the primary health care level like health centres and posts had a reducing effect on CHE [30, 49]. In some countries, seeking care from a private health facility is associated with increased CHE relative to seeking care at public facility [50], while in others, seeking care from public and private is associated with higher CHE compared to confessional structures [39]. Seeking services from traditional healers due to cultural beliefs on various illnesses is associated with high CHE [51].

#### Type of illness

The presence of a household member with a chronic disease increases the likelihood of experiencing CHE [44, 51, 52]. While the number of illness episodes amongst adults significantly increases the odds of CHE; the average number of illness episodes amongst children in a household has no effect on CHE [44]. Simple illness like coughs did not increase the risk of CHE [53]. In cases of TB care, households with an HIV patient are more likely to incur CHE than those not affected by HIV [50]. Contrary to expectation, having a disability has no effect on CHE [44]. However, occurrence of adverse events such as accidents or injury increases the likelihood of CHE [30, 53].

#### Characteristics of household members

Characteristics of the household head and members were mentioned in the majority of studies. However, different studies focused on different parameters including age of the household member, employment status, education level and female/male headed households. Households with older heads and older main income earners, lower education or with unemployed heads are more likely to incur CHE [52, 53]. Full-time employment is protective against CHE, especially amongst couples where the women has a full-time job [45]. Also, employment status and occupation are associated with CHE, for instance, having a household head who is a manual labourer increases the likelihood of CHE [16, 51, 52].

There are studies with different results, for instance, a study in Zambia shows that the education and employment status of the household head is not significantly associated with the likelihood of incurring CHE [49]. Also, a study in Nigeria finds counter intuitive evidence that more educated households are more likely to incur CHE than the less educated household [43].

There are mixed results about the probability of incurring CHE and gender. Female-headed households have a higher probability of facing CHE [47]. On the contrary, a study in Botswana observed that female-headed households are less likely to incur CHE [16]. In another study in Nigeria, households with a male patient are more likely to experience CHE [50], whereas in another study in Côte d’Ivoire, households of HIV-infected women have a higher risk of incurring CHE [54].

#### Geographical location and distance to health facility

Location of residence is seen as an important predictor of CHE. However, this varies by the location of the study. In Kenya, for instance, households located in marginalised counties have higher odds of incurring CHE [52], while in Benin, a study on TB patients shows the odds of CHE are higher for patients residing in urban areas, but when confounded with education, the effects disappear [40]. A study in Nigeria conducted amongst patients at a rural hospital shows that urban residents incur higher rates of catastrophic payments; this is due to transportation costs to the rural hospital [50]. Generally, living in urban areas is protective of CHE [30, 48, 55, 56]. However, it is found to be protective for non-poor, but not for the poor [47]. Living far from the nearest health care centre is associated with increased CHE [49, 55].

#### Social insurance/welfare scheme

Informal financing mechanisms through mutual organisations, informal groups and merry go rounds unlike formal health insurance is observed to reduce the risk of CHE [43]. Thus, patients with a poor social network are more likely to incur CHE [40]. Households that are enrolled in health insurance are engaged in mutual health organisations, or an informal social safety net (such as membership in a merry go round) have a reduced risk of catastrophic spending [30, 53].

In certain cases, health insurance is not a significant determinant, for instance, in Kenya, because it only covers a small proportion of households and only inpatient services [48]. Health insurance is observed not to protect households from CHE due to HIV/ART services. A study in Côte d’Ivoire observed no association between CHE and households having health insurance. This is because households continue to cope with HIV-related costs over time, thus, the financial burden increases [54].

#### Household size and composition

Households size is associated with CHE, with larger households (of more than five members) having a higher risk of incurring CHE [16, 51, 52, 54].

It is observed that having an elderly member (above 65 years) in the household imposes a higher risk of CHE for the household, meaning that elderly people are more vulnerable [39, 47, 52]. If the household has an elderly patient, (older than 40 years) CHE is likely to be high [40, 50]. In Nigeria, there seems to be a positive but not significant elderly effect [43]. Despite children being vulnerable to diseases, a study in Kenya showed that having a member aged under five decreased the odds of CHE [39].

### Household impoverishment in Sub Saharan-Africa countries

Household impoverishments due to catastrophic health expenditure are measured using different poverty lines in the different studies including the subsistence poverty line, the national poverty line (NPL) and the international poverty line (IPL). A study in Uganda that used both the national ($1.31) and the international ($1.25) poverty line [57] finds that the percentage poverty head count after health payment is higher when IPL is applied at 18.1%, while that of the NPL was 17.1%. Only one study in Malawi assesses impoverishment due to CHE for chronic illness [58]; all the other assessed impoverished due to CHE related to general health care.

**Fig. 4:**
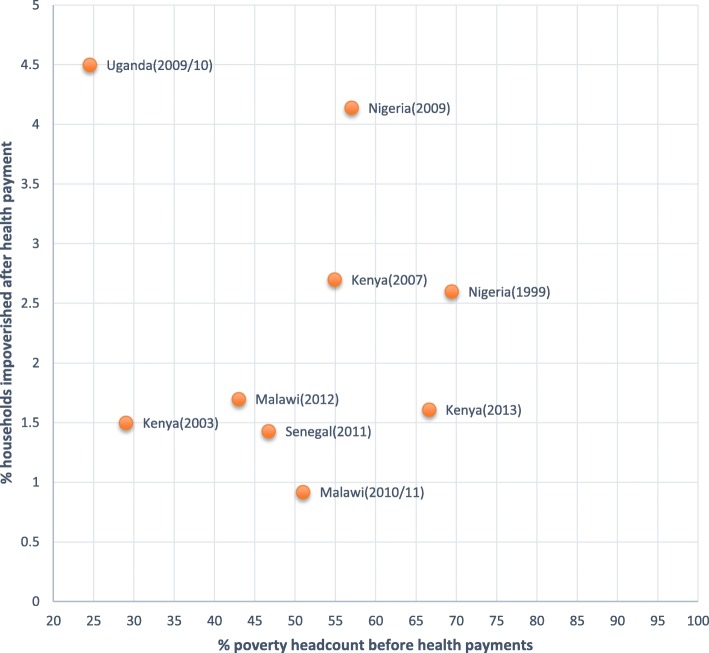
Level of impoverishment. The figure represents the percentage of household impoverished after health payments against the poverty head count prior to health payments across countries

The percentage of households that is impoverished ranges from 1.4 to 4.5% in the various countries. On average, 2% of households are impoverished due to health payments across all countries with Nigeria and Uganda having the largest proportion of household impoverished, 4.1% and 4.5% respectively. We note that the proportion of household impoverished in some instances increased and also decreased over time across countries and also within the countries. This is, for example, the case of Kenya in 2003, percentage of households impoverished was 1.5%, which increased in 2007 to 2.7% and decreased in 2013 to 1.6%. In Nigeria, a study using data collected in 1999 showed 2.5% households being impoverished and 4.1% in 2009, and in Malawi, households being impoverished were 0.9% in 2011 and 1.7% in 2012. Out of pocket payments induced a further 5.6% (ranging from 2–7%) of households on average into poverty with Uganda being the highest and an outlier at 18%. We observe no regional variations, but within regions there are variations, for instance, in West Africa, a study in 2009 [36] found that 4.1% of households in Nigeria were impoverished due to OOP relative to 1.4% of households observed in 2011 in Senegal [30]. In East Africa, similar variations were observed, a study in Uganda showed that 4.5% of households were impoverished [57] compared to neighbouring Kenya where 2.7% [59] and 1.6% [52] households were impoverished in 2007 and 2013 respectively Fig. 4. Table 3 summarises the pre- and post-poverty head count after health care payment and the associated poverty incidence.

**Table 3:** Impoverishment due to health payments in SSA by regions

Author	County	Pre-payment poverty head count (%)	Post-payment poverty head count (%)	Households impoverished (%)	Relative difference (%)
Xu et al., 2006 [47, 48]	Kenya	29^**α**^	30.5	1.5	5
Kwesiga et al., 2015 [57]	Uganda	24.5^**β**^	29	4.5	18
Barasa et al., 2017 [52]	Kenya	66.6^**β**^	68.21	1.61	2
Ichoku and Fonta, 2009 [41]	Nigeria	69.4^**β**^	72	2.6	4
Chuma and Maina, 2012 [59]	Kenya	54.9^**β**^	57.6	2.7	5
Sene and Cisse, 2015 [30]	Senegal	46.71^**β**^	48.14	1.43	3
Mchenga et al., 2017 [37]	Malawi	50.98^**β**^	51.9	0.92	2
Ichoku et al., 2009 [36]	Nigeria	57^**β**^	61	4.14	7
Wang et al., 2016 [58]	Malawi	43^ϕ^	44.7	1.7	4

^α^Subsistence poverty line

^β^National poverty line

^ϕ^International poverty line [$1.25 per day]

## Discussion

We observed some limitations that should be considered when interpreting the findings. First, the studies utilised different survey data including national household surveys, targeted population surveys and hospital data. Secondly, there were variations in the measurement of expenditure with some studies including only direct medical costs while others assessed both direct and indirect medical costs [52]. Also, there were variations of the thresholds applied across the different studies to measure catastrophic health expenditure, which makes it challenging to draw direct country comparisons. Furthermore, the proportion of households that experience CHE is dependent on the threshold used to define it [25].

In addition, impoverishment was measured using different poverty lines including subsistence, national and international poverty line. Given the main aim was to focus on CHE studies, the articles assessed on impoverishment were not exhaustive of the available literature on the same in SSA countries, but a representation of those that assessed both CHE and impoverishment.

We note that several studies rely on data collected several years (up to 10 years) back before the article was published, thus not providing a true reflection of the current context. Furthermore, use of alternative data means that the data was not solely collected for the purpose of this type of analysis, thus could bias the results.

The search and selection process was mainly conducted by the lead author. This could lead to a limitation or bias in the information retrieved from the articles selected for final review. However, all authors were involved in deciding the key search words, and the search string was discussed and agreed upon by the three reviewers. At every stage of the selection process, the three authors held frequent discussions to analyse the output(s). In addition, the use of MESH terms for key journal searches like PubMed ensured that all possible words were included in the search. We note that this has no substantial impact on the findings given the final articles that were reviewed represented 17 Sub-Saharan African countries from across all the different regions and a range of health areas.

West African countries incurred higher CHE relative to the other regions. This could be because most studies utilised convenient sampling of pre-selected vulnerable groups with small sample sizes rather than national representative household economic surveys, which were largely used in other regions. Furthermore, it has been argued that the use of convenience sampling is likely to bias results and conclusions; thus, interpretation should be done with caution [28]. For instance, of the 11 studies conducted in Nigeria, only two [41, 42] utilised national representative household surveys, and we note that most studies reported different incidences of CHE.

Patients with HIV/ART, TB and malaria experience the highest incidence of catastrophic expenditure. This could largely be due to the fact that individuals with HIV continue to incur health expenses throughout the time of their illness, while those with TB are in continuous medication for about 6 months or more, and Malaria could have several repeat episodes within a family.

Studies have found that affordability of treatment in LMICs is low as large proportions of population are pushed into poverty due to medicine procurement, hence the need for subsidies [60]. However, this review revealed that non-medical related costs like transportation costs which are invariably greater for the poor living far from the health facilities, food related costs, non-routine tests and inadequate care (due to shortages of drugs and medical services) in public primary health care facilities largely influence CHE which is consistent with other studies [54, 55, 61, 62]. This therefore means that on the contrary, subsidising the cost of drugs or removal of user fees alone may not necessarily protect households from CHE. It is revealing that non-medical expenditures are much higher than medical expenditures, with food and transport being the two most significant expenditure components [38]. We note that where user fees are abolished, CHE declines for the non-poor but surprisingly remains the same for the poor, thus not encouraging the poor to seek care [47].

All study findings are consistent that the poor have a higher incidence and are more likely to incur CHE than the well-off. Furthermore, studies show that the poor are more burdened with out of pocket payments and catastrophic expenditure [59, 63–65]. This is largely due to the fact that for households with a low income, even a small amount of health care costs can be catastrophic [49]. This is contrary to studies in low- and middle-income countries elsewhere like Asia, whereby the well-off are seen to have a higher incidence of CHE given their likelihood to spend more on health care unlike the poor [8, 66, 67]. This demonstrates that there is significantly less financial protection going to the poorest sections of the population in Sub-Saharan African countries.

Surprisingly, seeking services from the public sector increases the risk of CHE, despite no or modest charges for public sector [68]. This is possibly because most people who seek services from public service providers are from lower-income quintiles. In most SSA countries, people who seek care in the private sector are more likely to be well-off, hence have the capacity to pay. Nevertheless, this is not the case in many other countries that show CHE to be higher amongst people seeking care in private hospitals. This could be another factor that may explain the relatively high incidence of CHE even where user fees have been removed, given the inadequate quality of services in public facilities (due to shortages of drugs and medical services), individuals are compelled to seek better care elsewhere [49].

The review underscores the role of the type of illness in CHE. Consistent with other studies that have shown the impact of non-communicable chronic illnesses [25, 69], this review also notes that chronic illnesses contribute to a high risk of CHE [70–72]. Putting into consideration that infectious disease like HIV, TB and malaria are highly prevalent in Africa and have the highest incidence of CHE. This potentially poses a double burden on the households that are affected by both, thus driving the incidence of CHE further up. We observe that the time on ART decreases the risk of CHE; meaning that, patients who can access continuous ART treatment can be more financially secure [45]. However, if the main income earner is the one affected, time on ART increases the risk of CHE [54]. Contrary to the notion of collaborative HIV/TB services, we note that in case of TB care, households with HIV patients are more likely to incur CHE than those not affected by HIV because of the double disease burden [50].

Unlike in developed countries where health insurance is protective of CHE, this review emphasises informal social networks and mutual organisation common in the African setup, which help households to cope with costs. However, the review is inconclusive about the effect of formal health insurance in reducing CHE in SSA. There are nuances on the size of the household as a predictor of CHE. Although a larger household size is associated with higher CHE, households with more working adults are less likely to incur CHE [53] perhaps supporting the economies of scale argument [43]. Furthermore, it is also noted that smaller households have an increased risk of CHE which reflects a smaller support network from which financial assistance can be sort [45]. Elderly members in the household are seen to increase the risk of CHE [30] unlike children less than 5 years despite both being vulnerable to illnesses. This could be due to the fact that the elderly tends to be also income earners, thus when ill, there is dual burden unlike children who are under care of an elder. This is consistent with findings in other studies [73].

Distance to the health facility is associated with an increased likelihood of CHE, highlighting the significance of distance in increasing cost of access to health care [49]. Households in rural areas are also seen to experience higher CHE relative to those in urban areas excluding slum dwellers. Similar findings are observed in studies in Vietnam, Thailand and Serbia [6, 73, 74].

We observe that women are more likely to incur CHE due to their low financial status [43, 54]. In addition, we note that domestic violence against women increases the likelihood of experiencing CHE [51], given women’s welfare is vital to the household and injustice against them affects their income contribution, health and well-being [51].

Contrary to the notion that health payments have a higher impact in countries where poverty is high [3], we observe variations in the level of impoverishments in relation to the poverty head count before health payments. For instance, in Uganda, the level of poverty before health care payment was low, but the proportion of households impoverished as a result of health payment was higher than in all other countries. The proportion of households impoverished was seen to increase over time across the various countries, with the rapidly increasing population in Africa where the majority live below the poverty line; more people could be pushed into poverty if the right financial protection measures are not put in place. It is inconclusive if impoverishment due to health care payments was permanent or transitory as no study in in this review provided for that. An answer to this could however be given using panel data which are only limited available at a national scale in SSA.

## Conclusions

Overall, we observe that CHE and impoverishment are pervasive across all Sub-Saharan African countries, and the magnitude varies across and within countries and over time. The factors that keep CHE higher vary across the countries and are seen to cut across various socio-economic and demographic characteristics including economic status, type of health care provider, type of disease, household size, geographical location and social support schemes/network.

### Implication for research

This review underscores the importance of studies that assess CHE in SSA, and we notice the increased interest in this area given the rise in number of studies over time. However, we observe that majority of the studies were cross sectional, thus not sufficient for overtime analysis. Further research in SSA would be more beneficial if panel data were utilised to facilitate continuous monitoring of trends and robust over-time analysis on CHE and impoverishment.

### Implications for policy

Social protection interventions in Africa have primarily focused on the supply side through subsidising drugs, removal of user fees or provision of free health care, and most recently, expansion of social insurance schemes. However, this review has shown that most of these do not necessarily protect households from CHE due to other related non-medical costs like transport and food. The review emphasised on the role of informal social networks which are common in Africa like merry go round/mutual organisations and, hence, the need to explore policy innovations through these social networks, like insurance packages for informal/mutual groups. This review further highlights specific illnesses that drive CHE. In light of this, it is paramount for SSA countries to consider comprehensive and integrated health financing policies that cut across diseases, as this could help to draw synergies and efficiencies across disease areas and deal with possible dual disease burden. In addition, this review has paid specific attention to groups that are not financially autonomous. The fact that CHE was seen to be higher amongst the poor is an indication that the measures put in place have not been effective in protecting the poor. Given the context, there is a need to strengthen the social protection policies such that they are more holistic and effective in protecting the most vulnerable of population from catastrophic and impoverishing effects of health payments.

### Additional files

Additional file 1: PRISMA Checklist. This is PRISMA (Preferred Reporting Items for Systematic review and Meta-Analysis) checklist with the recommended items addressed in the review. (DOCX 26 kb)

Additional file 2: Search string. This is a summary of the search chain applied to search for articles in the various databases. (DOCX 77 kb)

Additional file 3: Quality assessment tool. This is the Quality Assessment Tool for Observational Cohort and Cross-Sectional Studies used to assess the quality of the articles included in this review. (DOCX 96 kb)

## Appendix 1

**Table 4:** Articles included in the review

No.	Title	Study questions/aims	Study location (country)	Study design	Sample size	Data source	Period of study	Health area
1.	(Xu et al. 2006b [48]) An empirical model of access to health care, health care expenditure and impoverishment in Kenya: learning from past reforms and lessons for the future	The impact of the Kenyan health financing system in the year 2003 on access to care and health spending. It will also shed light on the extent to which the prevailing system impoverished the population.	Kenya	Cross sectional	8407 households 38,009 individuals	Kenya health expenditure and utilisation survey 2003	2003	General health care
2.	(Onwujekwe et al. 2010 [65]) Are malaria treatment expenditures catastrophic to different socio-economic and geographic groups and how do they cope with payment? A study in southeast Nigeria	To determine the inequities in the household income depletion resulting from malaria treatment expenditures, the sacrifice of basic household needs [catastrophe] and the differences in payment strategies amongst different socio-economic and geographic groups in southeast Nigeria	Nigeria	Cross sectional	2250 households	HH survey	Not reported	Malaria
3.	(Kwesiga et al. 2015 [57]) Assessing catastrophic and impoverishing effects of health care payments in Uganda	To assess the catastrophic and impoverishing impact of paying for health care out of pocket in Uganda	Uganda	Cross sectional	6800 households	Uganda national HH survey 2009/2010	2009–2010	General health care
4.	(Akazili et al. 2017 [64]) Assessing the catastrophic effects of out-of-pocket healthcare payments prior to the uptake of a nationwide health insurance scheme in Ghana	To assess the catastrophic effect of OOP health care payments in Ghana to highlight the extent to which the health system protects HHs from the financial consequences of paying OOP for health services.	Ghana	Cross sectional	8687 households 36,488 individuals	Ghana living standard survey 2005/2006	2005–2006	General health care
5.	(Laokri et al. 2014 [40]) Assessing the economic burden of illness for tuberculosis patients in Benin: determinants and consequences of catastrophic health expenditures and inequities	To measure the risk, causes and consequences of catastrophic expenditures for tuberculosis and investigated potential inequities.	Benin	Cross sectional	250 TB patients	HH Survey	2008–2009	TB
6.	(Barasa et al. 2017 [52]) Assessing the impoverishing effects, and factors associated with the incidence of catastrophic health care payments in Kenya	The objectives of this study are to (1) examine the incidence and intensity of catastrophic health expenditures, (2) to examine the impoverishing effect of OOP health spending, and, (3) to explore factors that are associated with catastrophic health spending in Kenya.	Kenya	Cross sectional	33,675 households	Kenya health expenditure and utilisation survey 2013	2013	General health care
7.	(Ichoku and Fonta, 2009 [41]) Catastrophic healthcare financing and poverty: empirical evidence from Nigeria	To analyse the incidence and severity of catastrophic healthcare financing using different definitions of catastrophic healthcare and to examine the links between this phenomenon and poverty.	Nigeria	Cross sectional	7667 households	General household surveys of the federal office of statistics	1999	General health care
8.	(Chuma and Maina, 2012 [59]) Catastrophic health care spending and impoverishment in Kenya	To estimates the burden of out-of-pocket payments in Kenya; the incidence and intensity of catastrophic health care expenditure and the effect of health spending on national poverty estimates.	Kenya	Cross sectional	8414 households	Kenya health expenditure and utilisation survey 2007	2007	General health care
9.	(Buigut et al. 2015 [53]) Catastrophic health expenditure and its determinants in Kenya slum communities	To examine the incidence and determinants of catastrophic health expenditure amongst urban slum communities in Kenya	Kenya	Longitudinal	8171 individuals	Indicator development for surveillance of urban emergency (IDSUE)	2011–2013	General health care
10.	(Su et al. 2006 [44]) Catastrophic household expenditure for health care in a low-income society: a study from Nouna District, Burkina Faso	To quantify the extent of catastrophic household health care expenditure and determine the factors responsible for it in Nouna district, Burkina Faso	Burkina Faso	Cross sectional	774 households	Nouna health district HH survey	2000–2001	General health care
11.	(Sene and Cisse, 2015 [30]) Catastrophic out-of-pocket payments for health and poverty nexus: evidence from Senegal	The purpose of this study is to cast light on the determinants of catastrophic household out-of-pocket health expenditures and to assess their implications on poverty.	Senegal	Cross sectional	17,891 households	Poverty monitoring survey 2011	2011	General health care
12.	(Dyer et al. 2013 [45]) Catastrophic payment for assisted reproduction techniques with conventional ovarian stimulation in the public health sector of South Africa: frequency and coping strategies	How often does out-of-pocket payment (OPP) for assisted reproduction techniques (ART) with conventional ovarian stimulation result in catastrophic expenditure for households?	South Africa	Experimental-prospective observational study	135 ART couples	Hospitals survey	2009–2011	HIV-ART
13.	(Brinda et al. 2014 [51]) Correlates of out-of-pocket and catastrophic health expenditures in Tanzania: results from a national household survey	To investigate the determinants influencing OOP health expenditure amongst the adult as well as the older population aged above 60 Years in Tanzania	Tanzania	Cross sectional	3265 households 8297 individuals	Tanzania national panel survey 2008/2009	2008–2009	General health care
14.	(Masiye et al. 2016 [49]) Does user fee removal policy provide financial protection from catastrophic health care payments? Evidence from Zambia	To examine the extent and patterns of financial protection from fees following the decision to abolish user fees in public primary health facilities.	Zambia	Cross sectional	12,000 households 60,000 individuals	Zambia health expenditure and utilisation survey 2014	2014	General health care
15.	(Arsenault et al., 2013 [55]) Emergency obstetric care in Mali: catastrophic spending and its impoverishing effects on households	To investigate the frequency of catastrophic expenditure for emergency obstetric care, explore its risk factors and assess the effects of these expenditures on households in the Kayes region, Mali	Mali	Case-control	484 women	HH survey	2008–2011	Obstetric care
16.	(Okoronkwo et al. 2015 [71]) Economic burden and catastrophic cost amongst people living with type2 diabetes mellitus attending a tertiary health institution in south-east zone, Nigeria	To assess the magnitude of economic burden borne and catastrophic costs incurred by PLWDs in Nigeria	Nigeria	Cross sectional	308 People living with disability (PLWD)	Hospitals survey	2011–2012	Diabetes
17.	(Ughasoro et al. 2014 [72]) Economic cost of treatment of childhood epilepsy in Enugu, southeast Nigeria	To determine the economic costs and the level of CHE due to childhood epilepsy	Nigeria	Cross sectional	134 Patients	Hospitals survey	2012	Epilepsy
18.	(Mills et al. 2012 [14]) Equity in financing and use of health care in Ghana, South Africa, and Tanzania: implications for paths to universal coverage	To report the results of a three-country study on the equity of health system financing and service use	Ghana; South Africa; Tanzania	Comparative study	Not reported	National HH survey	2008	General health care
19.	(Onwujekwe et al. 2012 [63]) Examining inequities in incidence of catastrophic health expenditures on different healthcare services and health facilities in Nigeria	To estimate the level of CHE for different healthcare and facilities and their distribution across socio economic status	Nigeria	Cross sectional	4473 households	HH survey	Not reported	General health care
20.	(Onwujekwe et al. 2009 [61]) Examining catastrophic costs and benefit incidence of subsidised antiretroviral treatment (ART) programme in south-east.	To examine the extent to which costs of subsidised antiretroviral treatment programmes are catastrophic and the benefit incidence that accrues to different population groups	Nigeria	Cross sectional	301 ART patients	Hospital database	Not reported	HIV-ART
21.	(Onwujekwe et al. 2016 [38]) Examining geographic and socio-economic differences in outpatient and inpatient consumer expenditures for treating HIV/AIDS in Nigeria	To provide information and the resultant incidence of CHE from medical and non-medical expenditures incurred on outpatient visits [OPV] from different social-economic groups and geographical conditions.	Nigeria	Cross sectional	1200 people living with HIV	HH survey	2013	HIV-ART
22.	(Akinkugbe et al. 2012 [16]) Health financing and catastrophic payments for health care: evidence from household-level survey data in Botswana and Lesotho	To assess the degree of inequality in the distribution of health expenditure across wealth quintiles in Botswana and Lesotho.	Lesotho; Botswana	Comparative study	6882 households (Lesotho) 6053 households (Botswana)	HH expenditure survey 2002/2003	2002–2003	General health care
23.	(Castillo-Riquelme et al. 2008 [68]) Household burden of malaria in South Africa and Mozambique: is there a catastrophic impact?	To evaluate treatment seeking behaviour financial impact and time lost due to malaria events in southern Mozambique and eastern South Africa	South Africa; Mozambique	Comparative study	827 households (South Africa) 828 households (Mozambique)	HH survey	2001–2002	Malaria
24.	[Ukwaja et al. 2013 [46, 50] Household catastrophic payments for tuberculosis care in Nigeria: incidence, determinants, and policy implications for universal health coverage	To investigate the incidence, intensity, distribution and correlates of catastrophic payments for TB care and policy implications for TB care and their primary care services	Nigeria	Cross sectional	452 TB patients	HH survey	2011	TB
25.	(Mchenga et al. 2017 [37]) Impoverishing effects of catastrophic health expenditures in Malawi	To Investigate the effect of catastrophic OOP on the incidence and depth of poverty in Malawi	Malawi	Cross sectional	12,271 individuals	Integrated household survey 2010/2011	2010–2011	General health care
26.	(Ichoku et al. 2009 [36]) Incidence and intensity of catastrophic healthcare financing and impoverishment due to out-of- pocket payments in southeast Nigeria	To examine incidence and intensity of catastrophic health care financing and the impoverishing effects, as well as equity concerns due to OOP for healthcare in Southeast Nigeria.	Nigeria	Cross sectional	1500 households	HH survey	Not reported	General health care
27.	(Ilunga-Ilunga et al. 2015b [39]) Incidence of catastrophic health expenditures for households: an example of medical attention for the treatment of severe childhood malaria in Kinshasa reference hospitals, Democratic Republic of Congo	To estimate the incidence of catastrophic health expenditures incurred by households in which one child suffered severe malaria and subsequently attended Kinshasa referral hospital.	Democratic Republic of Congo	Cross sectional	1350 children	HH survey	Not reported	Malaria
28.	(Adisa, 2015 [43]) Investigating determinants of catastrophic health spending amongst poorly insured elderly households in urban Nigeria	To investigate the key determinants of CHE amongst poorly insured elderly households in Nigeria.	Nigeria	Cross sectional	1176 households	Nigerian general HH panel survey 2010	2010	General health care
29.	(Cleary et al. 2013 [56]) Investigating the affordability of key health services in South Africa	To identify Characteristics of households that experience difficulties in affording health care	South Africa	Cross sectional	3727 patients	Exit interviews	Not reported	Obstetric care/TB/ART
30.	(Ataguba, 2012 [42]) Reassessing catastrophic health-care payments with a Nigerian case study	What might constitute fair indices of catastrophic payment, which explicitly recognise diminishing marginal utility of income as reflected in some principle of vertical equity? This paper aims to examine such indices and how best to assess them.	Nigeria	Cross sectional	19,518 households	Nigerian national living standard survey 2003/2004	2003–2004	General health care
31.	(Counts and Skordis-Worrall, 2016 [70]) Recognising the importance of chronic disease in driving healthcare expenditure in Tanzania: analysis of panel data from 1991 to 2010	This study compares the level and predictors of expenditure on healthcare between chronic disease-affected (CDA) and unaffected (CDU) households in this region using 19-year panel data.	Tanzania	Longitudinal	900 households 6353 individuals	Modelled data–Kagera health development survey	1991–2010	Chronic disease
32.	(Wang et al. 2016 [58]) The economic burden of chronic non-communicable diseases in rural Malawi: an observational study	To estimate both the HH direct, indirect and total costs due to CNCDs; and the economic burden households bear as a result of these costs in Malawi	Malawi	Cross sectional	1199 households 5643 individuals	HH survey	2012	Chronic non-communicable
33.	(Beaulière et al. 2010 [54]) The Financial burden of morbidity in HIV-infected adults on antiretroviral therapy in Côte d’Ivoire	To estimate the financial burden of health care for households with HIV-infected adults taking antiretroviral therapy (ART]) in Côte d’Ivoire.	Côte d’Ivoire	Cross sectional	1190 adults	HH survey	2007	HIV-ART
34.	(Xu et al., 2006a [47]) Understanding the impact of eliminating user fees: utilisation and catastrophic health expenditures in Uganda	Examine changes in utilisation and catastrophic health expenditure	Uganda	Cross sectional	6655 households 33,988 individuals	Social economic survey 1997, 2000, 2003	1997, 2000, 2003	General health care

## Appendix 2

**Table 5:** Determinants of catastrophic health expenditure

No.	Author	County	Health area	Determinants						
				Household economic status	Type of health provider	Type of illness	Household member characteristics	Geographical location	Social insurance/health scheme	Household size and composition
1	(Xu et al. 2006 [47, 48])	Kenya	General health care	Higher income (−)	Public facilities (+) inpatient services (+)	Reported Illness (+)	Employment (−) Education (−)	Urban area (−)	Health insurance (NS)	Under 5 (−)
2	(Laokri et al. 2014 [40])	Benin	TB	Lower quintile (+)	NA	Adverse pre-diagnosis (+)	Education (+) gender(NS)	NA	Poor social network (+) health insurance (+)	Small household (+) above 40 years (+)
3	(Barasa et al. 2017 [52])	Kenya	General health care	Poorest quintile (+)	NA	Chronic disease (+)	Unemployed (+)	Marginalised location (+)	NA	Older HH head (+) large HH size (+)
4	(Buigut et al. 2015 [53])	Kenya	General health care	NA	Public hospital (+)	Injury (+) simple illness, e.g. cough (−)	Working adults (−)	Slums (+)	Safety net (−)	Older income earner above 55 years (+)
5	(Su et al. 2006 [44])	Burkina Faso	General health care	Higher quintile (NS)	NA	illness episodes (+) chronic illness (+) disabled (NS)	NA	NA	NA	HH size (+)
6	(Sene and Cisse, 2015 [30])	Senegal	General health care	NA	Health centre/posts (+)	Accidents (+)	NA	Rural areas (+)	Health insurance (−)	Elderly members (+)
7	(Dyer et al. 2013 [45])	South Africa	HIV-ART	Poorest quintile (+)	NA	Pre-ART treatment (−)	Education level (+) employment (−) age (NS)	NA	Medical scheme (NS)	Larger HHs (−)
8	(Brinda et al. 2014 [51])	Tanzania	General health care	Low socio-economic (+) low HH asset (+)	Traditional healer (+)	Chronic disease (+)	Manual labourer (+)	NA	NA	HH size above 5 (+)
9	Masiye et al., 2016 [49])	Zambia	General health care	Rich wealth quintile (−)	Primary health care facility (−)	NA	Education attainment (NS) employment status (NS) sex (NS)	Distance to health facility (+) region of residence (NS)	NA	NA
10	(Arsenault et al. 2013 [55])	Mali	Obstetric care	Poorest (+)	NA	NA	No education (+)	Remote community (+) 40 KM away from health facility (+)	NA	NA
11	(Akinkugbe et al. 2012 [16])	Botswana, Lesotho	General health care	NA	NA	NA	Unemployed HH head (+) female headed HHs (−) educated head (−)	Rural areas (+)	NA	HH size (+) HH member above 65 years (+)
12	(Ukwaja et al. 2013 [46, 50])	Nigeria	TB	NA	Private facility (−)	HIV positive status (+) TB smear positive (+)	Formal education (+) primary income earner (+)	Urban residence (−)	NA	Above 40 years (+)
13	(Ilunga-Ilunga et al. 2015 [39])	Democratic Republic of Congo	Malaria	Poor and Middle income (+)	Private hospital (+)	Clinical Malaria (+)	Female headed HHs (+)	NA	NA	NA
14	(Adisa, 2015 [43])	Nigeria	General health care	Higher income (−)	NA	NA	Educated (+) female age (+)	NA	Informal health financing (−) non-enrolment in insurance (+)	NA
15	(Cleary et al. 2013 [56])	South Africa	Obstetric care/TB/ART	NA	NA	Obstetrics patients (+)	Employment (−) education (+)	Urban areas (−)	NA	NA
16	(Counts and Skordis-Worrall, 2016 [70])	Tanzania	Chronic disease	NA	NA	NA	Education level (+) male HH head (−)	Urban areas (+)	NA	HH size (+) number of adults (+)
17	(Beaulière et al. 2010 [54])	Côte d’Ivoire	HIV-ART	Higher income (−)	NA	Time spent on ART (−)	Education level (−) female HIV patients (−)	NA	Health insurance (NS)	HH size (−)
18	(Xu et al. 2006a [47])	Uganda	General health care	HH income (NS)	Private facilities (+)	NA	Low education (+) male HHs (−)	Urban areas (−)	NA	HH with over 65 yrs. (+)

*HH* Household, *NA* not applicable, *NS* not significant, (−) decreasing odds/likelihood, (+) increasing odd/likelihood

## Appendix 3

**Table 6:** Data extracted from articles: Incidence and intensity of CHE and impoverishment reported in various articles

No.	Author	County	Primary outcome	Threshold	Incidence range	Intensity range	Poverty Line	Poverty Head count	HHs Impoverished	Out of Pocket expenditure	Type of costs	Inpatient & Outpatient costs
1	(Xu et al., 2006) [47, 48]	Kenya	CHE & Impoverishment	40% – Non-food Expenditure	40% (4.1)	NR	$0.5 (28 KES)	30.5	1.5%	Direct costs	Medical & Non-Medical	Inpatient & Outpatient
2	(Onwujekwe et al., 2010) [66]	Nigeria	CHE	5% - Non-food expenditure	5% (8.2)	NR	NR	NR	NR	Direct costs	Medical & Non-Medical	Outpatient
3	(Kwesiga et al., 2015) [57, 59]	Uganda	CHE & Impoverishment	10% - HH income	5%, 10%, 15%, 25% (38, 22.8, 15.3, 6.7)	5%, 10%, 15%, 25% (3.8, 2.5 1.7, 0.8)	$1	29%	4.5%	Direct costs	Medical	Outpatient
4	(Akazili et al., 2017) [65]	Ghana	CHE	5% - 20% HH expenditure 10% - 40% Non-food expenditure	5%,10%,15%,20% - HH Income, (11, 5.16, 3.39,2.56) 10%, 20%, 30%, 40% - Non-food expenditure (10.7, 4.91, 3.17, 2.43)	5%,10%,15%,20% - HH Income, (1.83, 1.47, 1.26, 1.11) 10%, 20%, 40% -Non-food expenditure (3.39, 2.68, 2.01)	NR	NR	NR	Direct costs	Medical	Inpatient & Outpatient
5	(Laokri et al., 2014) [40]	Benin	CHE	10% - HH income	5%, 10%, 15%, 20%, 25% (88.6, 71.8, 58, 45.7, 36.3)	5%, 10%, 15%, 20%, 25% (12.8, 7.8, 2.8, 2.2, 7.2)	NR	NR	NR	Direct costs	Medical	Outpatient
6	(Barasa et al., 2017) [52]	Kenya	CHE & Impoverishment	40% – Non-food Expenditure	40% (6.58)	40% (5.73)	$0.75 (29.13 Urban, 15.62 Rural per month)	68.21	1.6%	Direct costs	Medical & Non-Medical	Inpatient & Outpatient
7	(Ichoku and Fonta, 2009) [41]	Nigeria	CHE & Impoverishment	10%, 30%, 40% - HH expenditure	2.5%, 5%, 10%, 15%, 20%, 30%,40% (47.8, 38.8, 22.7, 13.6, 9, 3.5, 1.7)	2.5%, 10%, 15%, 20%, 30%,40% (6.2, 5.8, 4.7, 3.6, 2.8, 1.5, 0.9)	$22.2 per month	72	2.6%	NR	NR	NR
8	(Chuma and Maina, 2012) [60]	Kenya	CHE & Impoverishment	10% - HH expenditure & 40% - Non-food expenditure	10%, 25%, 40% (15.5, 16, 11.4)	10%, 25%, 40% (11, 27.2, 25.4)	$0.5 (1257 KES per month)	57.6	2.7%	Direct costs	Medical	Inpatient & Outpatient
9	(Buigut et al., 2015) [53]	Kenya	CHE	10% HH income	10%, 15%, 20%, 30% (22.8, 4.11, 2.7, 1.55)	NR	NR	NR	NR	Direct costs	Medical & Non-Medical	Outpatient
10	(Su et al., 2006) [44]	Burkina Faso	CHE	40% – Non-food Expenditure	20%, 30%, 40% (15.12, 10.59, 8.66)	NR	NR	NR	NR	Direct costs	Medical & Non-Medical	Inpatient & Outpatient
11	(Sene and Cisse, 2015) [30]	Senegal	CHE & Impoverishment	10% - HH expenditure	5%, 10%, 15%,20%, 25% (16.2, 6.26, 2.33, 1.38, 0.87)	NR	$1	48.14	1.4%	Direct costs	Medical & Non-Medical	Inpatient & Outpatient
12	(Dyer et al., 2013) [45]	South Africa	CHE	40% – Non-food Expenditure	40% (22)	NR	NR	NR	NR	Direct & Indirect costs	Medical & Non-Medical	Outpatient
13	(Brinda et al., 2014) [51]	Tanzania	CHE	40% – Non-food Expenditure	40% (18)	NR	NR	NR	NR	Direct costs	Medical	Inpatient & Outpatient
14	Masiye et al., 2016) [49]	Zambia	CHE	10% - HH income & 40% - Non-food expenditure	10%, 40% (11.2, 9.3)	NR	NR	NR	NR	Direct costs	Medical & Non-Medical	Outpatient
15	(Arsenault et al., 2013) [55]	Mali	CHE	10% - HH income	5%, 10%, 15% (53.5, 33.5, 20.7)	NR	NR	NR	NR	Direct costs	Medical & Non-Medical	Outpatient
16	(Okoronkwo et al., 2015) [72]	Nigeria	CHE	30% – Non-food Expenditure	30% (45)	NR	NR	NR	NR	Direct costs	Medical & Non-Medical	Outpatient
17	Ughasoro et al., 2014) [73]	Nigeria	CHE	40% – Non-food Expenditure	40% (Inpatient-63.6, Outpatient 34.1)	NR	NR	NR	NR	Direct costs	Medical & Non-Medical	Inpatient & Outpatient
18	(Mills et al., 2012) [14]	Ghana; South Africa; Tanzania	CHE	40% – Non-food Expenditure	40% (Ghana-2.43, Tanzania-1.52, South Africa-0.09)	NR	NR	NR	NR	Direct costs	NR	NR
19	(Onwujekwe et al., 2012) [64]	Nigeria	CHE	40% – Non-food Expenditure	5%, 40% (57, 27)	NR	NR	NR	NR	Direct costs	Not indicated	Inpatient & Outpatient
20	(Onwujekwe et al., 2009) [62]	Nigeria	CHE	40% – Non-food Expenditure	40% (9.8)	NR	NR	NR	NR	Direct costs	Medical & Non-Medical	Outpatient
21	(Onwujekwe et al., 2016) [38]	Nigeria	CHE	40% – Non-food Expenditure	10%, 40% (Inpatient-100, 40.3: Outpatient-94.3, 7.7)	NR	NR	NR	NR	Direct costs	Medical & Non-Medical	Inpatient & Outpatient
22	(Akinkugbe et al., 2012) [16]	Botswana, Lesotho	CHE & Impoverishment	20% Non-food Expenditure & 40% – Non-food Expenditure	20%, 40% (Botswana-11.1, 3.22: Lesotho-7.43, 1.25)	NR	NR	NR	NR	Direct costs	Medical & Non-Medical	Not indicated
23	(Castillo-Riquelme et al., 2008) [69]	South Africa	CHE	10% - HH income & 40% - Non-food expenditure	10%, 40% (Mozambique-32, 34; Kwazulu Natal-11.4, 12.5; Mpumalanga-10.2, 9)	NR	NR	NR	NR	Direct & Indirect costs	Medical	Inpatient & Outpatient
24	(Ukwaja et al., 2013) [46, 50]	Nigeria	CHE	10% - HH income & 40% - Non-food expenditure	10%, 15%, 25%, 40% (65, 84, 68, 44)	10%, 15%, 25%, 40% (6, 12.3, 10.7, 8.3)	NR	NR	NR	Direct costs	Medical & Non-Medical	Outpatient
25	(Mchenga et al., 2017) [37]	Malawi	CHE & Impoverishment	10% - Non-food expenditure & 40% - Non-food expenditure	10%, 20%, 30%, 40% (9.37, 3.41, 1.6, 0.7)	10%, 20%, 30%, 40% (1.01, 0.43, 0.2, 0.08)	$0.6 (K37002 Per annum)	51.9	0.9%	Direct costs	Not Indicated	Not indicated
26	(Ichoku et al., 2009) [36]	Nigeria	CHE & Impoverishment	5% - HH expenditure & 10% HH expenditure	5%, 10% (29, 21.75)	5%, 10% (5.67, 4.4)	$1 (N2900 per month)	61	4.1%	Direct costs	Medical & Non-Medical	Outpatient
27	(Ilunga-Ilunga et al., 2015) [39]	Democratic Republic of Congo	CHE	10% - HH expenditure & 40% - Non-food expenditure	10%, 40% (65.3, 94.7)	NR	NR	NR	NR	Direct costs	Not Indicated	Inpatient
28	(Adisa, 2015) [43]	Nigeria	CHE	10% - HH expenditure	10% (9.61)	NR	NR	NR	NR	Direct costs	Medical	Inpatient & Outpatient
29	(Cleary et al., 2013) [56]	South Africa	CHE	10% - HH expenditure	10% (All 39.35 ~ TB-32.9, ART- 22.7, CEOC-66.1)	NR	NR	NR	NR	Direct costs	Medical & Non-Medical	Inpatient & Outpatient
30	(Ataguba, 2012) [42]	Nigeria	CHE	10% - HH expenditure & 40% - Non-food expenditure	10%, 15%, 20%, 40% (25.4, 19.6, 15.6, 17.2)	10%, 15%, 20%, 40% (6.01, 4.9, 4.02, 4.9)	NR	NR	NR	Direct costs	Medical	Not Indicated
31	(Counts and Skordis-Worrall, 2016) [71]	Tanzania	CHE	40% – Non-food Expenditure	40% (Affected-7.5, Un affected - 6.7)	NR	NR	NR	NR	Direct costs	Medical & Non-Medical	Inpatient & Outpatient
32	(Wang et al., 2016) [58]	Malawi	CHE & Impoverishment	10% - Non-food expenditure & 40% - Non-food expenditure	10%, 25%, 40% (21.3, 10.7, 4.5)	NR	$1.25	44.7	1.7%	Direct costs & Indirect costs	Medical & Non-Medical	Outpatient
33	(Beaulière et al., 2010) [54, 74]	Côte d'Ivoire	CHE	40% – Non-food Expenditure	10%, 20%, 40% (50, 28, 12)	NR	NR	NR	NR	Direct costs	Medical & Non-Medical	Outpatient
34	(Xu et al., 2006a) [47]	Uganda	CHE	40% – Non-food Expenditure	40% (1997-4.82, 2000-3.15, 2003-2.92)	NR	NR	NR	NR	Direct costs	Medical	Not indicated

## Abbreviations

ART: Antiretroviral therapy
AU: African Union
CHE: Catastrophic health expenditure
HIV: Human immunodeficiency virus
LMIC: Low- and middle-income countries
OOP: Out of pocket
PRISMA: Preferred Reporting Items for Systematic Reviews and Meta-Analyses
SSA: Sub-Saharan Africa
TB: Tuberculosis
UHC: Universal Health Coverage
WHO: World Health Organization
