# Supplementary material for: Understanding variations in catastrophic health expenditure, its underlying determinants and impoverishment in Sub-Saharan African countries: a scoping review
Source: Syst Rev. 2018 Sep 11;7:136. doi: 10.1186/s13643-018-0799-1 (PMC6134791; doi:10.1186/s13643-018-0799-1)
Supplement: Supplementary file 2 — Search string. This is a summary of the search chain applied to search for articles in the various databases. (DOCX 77 kb) [file 13643_2018_799_MOESM2_ESM.docx]

**Additional file 2: Search string for various databases**

| **PUBMED**  ((((((((catastrophic) OR impoverishment) OR financial burden) OR economic burden) AND expenditure, health[MeSH Terms]) OR cost, healthcare[MeSH Terms]) AND africa south of the sahara[MeSH Terms]) AND full text[sb] AND "last 10 years"[PDat] AND Humans[Mesh] AND English[lang])  **JSTOR**  (((((((Catastrophic) OR (impoverish*)) OR ("economic burden")) OR ("financial burden")) AND ("health expenditure")) OR ("healthcare cost*")) AND (Africa)) AND la:(eng OR en) AND disc:(healthsciences-discipline OR health-discipline)  **SUBJECT:** Health policy; Health science  **Article type** -  Articles ; Reviews**;** Books; Research Reports  **EBSCO host**  TX catastrophic OR TX impoverish* OR TX "financial burden" OR TX "economic burden" AND TX "health expenditure" OR TX "healthcare cost" AND TX Africa  CINAHL, Econlit, PsycINFO  **Limiters** - Published Date: 20060101-20170531  **Narrow by Language:** - english  **Narrow by Subject:** - health: government policy; regulation; public health  **Search modes** - Boolean/Phrase  #####No limiters per journal  **WEB OF SCIENCE**  TS=((catastrophic OR Impoverish* OR financial burden OR economic burden) AND (health expenditure OR healthcare costs) AND Africa)  **Timespan:** 2006-2017. **Indexes:** SCI-EXPANDED, SSCI, A&HCI, ESCI. |
| --- |
